# Investigating the electronic properties and reactivity of polyaniline emeraldine base functionalized with metal oxides

**DOI:** 10.1038/s41598-024-72435-7

**Published:** 2024-11-06

**Authors:** Rania Badry, Hanan Elhaes, Asmaa Ibrahim, Ahmed Refaat, Medhat A. Ibrahim

**Affiliations:** 1 Physics Department, Faculty of Women for Arts, Science and Education, Ain Shams University, Cairo, 11757 Egypt; 2 Spectroscopy Department, National Research Centre, 33 El-Bohouth St., Dokki, Giza, 12622 Egypt; 3 Molecular Modeling and Spectroscopy Laboratory, Centre of Excellence for Advanced Science, National Research Centre, 33 El-Bohouth St., Dokki, Giza, 12622 Egypt

**Keywords:** B3LYP/SDD, PANi, TDM, HOMO–LUMO, Global reactivity descriptors, Alkali and heavy metal oxides, Materials science, Physics

## Abstract

Due to its appealing qualities, such as its miniature size and the ability to modify physical properties through chemical synthesis and molecular design, polymer material offers considerable advantages over traditional inorganic material-based electronics. Conjugate polymers are particularly interesting because of their molecular design capabilities, which enable the synthesis of conducting polymers with a variety of ionization potentials and electron affinities (EA), and their ability to control the energy gap and electronegativity (χ). Accordingly, density functional theory (DFT) at the B3LYP/SDD model was used to present possible interactions between polyaniline (PANi) and both alkali and heavy metal oxides. Total dipole moment (TDM), HOMO–LUMO band gap energy (ΔE), ionization energy (IE), EA, chemical hardness (η), chemical potential (μ), electrophilicity index (ω), chemical softness (S), and χ are calculated. TDM of PANi increased while ΔE decreased due to functionalization. The distribution of electronic charge density in molecular electrostatic potential (MESP) maps together with the results of ω reflected the electrophilic nature. The obtained results confirmed that the addition of metal oxides significantly improves the TDM, ΔE, and reactivity descriptors. A strong correlation between the experimental and calculated IR spectra was observed. Additionally, PANi–2MgO and PANi–2MnO model molecules exhibited the highest reactivity. Accordingly, PANi functionalized with MgO and MnO are promising candidates for energy storage devices.

## Introduction

In the global context, energy has emerged as a key area of study for sustainable development. It is now imperative that more effective energy storage systems or devices be developed and refined^1,2^. In response to the increasing need for renewable and sustainable energy sources, efforts have been made to produce energy storage systems that are lightweight, eco-friendly, and flexible. Thus, energy storage systems that are clean, effective, and able to meet present energy needs are the focus of current research^3–5^. High energy density, high power, lightweight, portable size, long shelf life, low cost, and other desired specifications should all be met by an energy storage device^6,7^.

The wide ranges of electrical conductivity, thermal stability, processability, and mechanical flexibility of conducting polymers are drawing a lot of interest^8^. Since they have so many uses across a wide range of industries, intrinsically conducting polymers have drawn the interest of scientists^9,10^. They are also referred to as synthetic metals because of their electrical conductivity values, which are comparable to those of metals^11^. The idea was to create an organic polymer that could compete with a metal’s electrical properties by doping it to increase its electric conductivity^12–14^. PANi, polyacetylene (PA), polythiophene (PTh), polypyrrole (PPy), and other compounds are examples of electrically conducting polymers^15–17^. Because of their high conductivity, high pseudo-capacitance, and simplicity in processing and manufacturing, conducting polymers have garnered a lot of interest. Nevertheless, their mechanical properties are poor, and they have a short life cycle^18,19^.

Due to the dark colour of its pigment, PANi is also known as aniline black. PANi structure consists of benzoid and quinoid rings^20,21^. In terms of oxidation states, it can be found in three different forms: fully reduced leucoemeraldine, which is made up of a benzoid ring; fully oxidized pernigraniline, which is made up of a quinoid ring; and partially oxidized and partially reduced emeraldine, which is made up of both a benzoid and a quinoid ring. Leucoemeraldine and pernigraniline are the two non-conducting forms among these three, while emeraldine is the conducting form^20,22,23^. Doping makes PANi more electrically conductive since the dopant material improves electrical conductivity by lowering PANi’s band gap. PANi is an essential conducting polymer in pseudocapacitor materials for electrochemical energy storage because it is lightweight, less toxic, eco-friendly, flexible, affordable, and exhibits thermal stability^24–26^.The application of PANi in supercapacitors is limited by their mechanical stability and tendency to degrade during repeated charge–discharge cycles, despite their high conductivity and ease of synthesis.

Interesting nanocomposites are typically produced when conducting polymers and metal oxides are combined, utilizing their respective advantages to develop and optimize a material at the nanoscale^19^. Materials with a nanoscale structure that enhance a product’s macroscopic qualities are called nanocomposites. Materials made of nanocomposites offer superior wear, long-term heat, and scratch resistance, as well as unique mechanical and barrier properties and weight reduction^27^. Increases in electrical breakdown strength, colour, melting temperature, charge capacity, magnetism, and interaction zone expansion are the reasons why conducting polymer nanocomposites differ from one another. In addition to the specific materials used, the morphology and interfacial properties of the mixture also affect the characteristics of nanocomposite electrodes^28^.

Umar et al. developed a method for synthesizing reduced graphene oxide nanosheets with PANi surface decoration, revealing their potential as advanced electrode materials for high-performance supercapacitors^29^. In another study, Güngör et al. synthesized cost-effective nanocomposites using PANi and copper zinc tin sulfide (Cu_2_ZnSnS_4_) material, resulting in supercapacitors with high cyclic stability, making them ideal for long-term energy storage applications^30^. Fayemi et al. synthesized carbon quantum dots (Cdots) from pencil graphite precursors using a bottom-up method and incorporated them into PANi to create a nanocomposite. The synthesized Cdots-PANi nanocomposite-modified screen-printed carbon electrodes (SPCE) demonstrated superior redox potentials, faster electron transfer kinetics, a larger surface area, and greater stability, indicating potential as sensitive electrochemical sensors^31^.

The benefits of conducting polymer-metal oxide nanocomposites include the durability and hardness of metal oxides combined with the toughness, flexibility, and coatability of polymers^29,32^. They also possess certain synergistic properties that set them apart from the constituent materials. The properties of energy storage devices, including specific surface area, electrical conductivity, ionic conductivity, cyclic stability, specific capacitance, and energy and power density, have been shown to be greatly enhanced by the use of nanocomposite electro-active materials^33–35^. As a result, conducting polymer-metal oxide nanocomposites are now a key component of the materials used in energy storage applications.

Furthermore, the band gap energy of materials used in solar cells, optoelectronics, and photonics is a significant factor in determining which wavelengths of light the material can absorb and convert to electrical energy. If the material’s band gap matches the wavelengths of light shining on the device, the device can make efficient use of all available energy. Photons with E ≥ E_g_ can absorb and generate photocurrent, while photons with E < E_g_ pass through the cell without raising the output load. Furthermore, when E exceeds E_g_, photons contribute to thermalization losses. This is because they have more energy than what is used to generate photocurrent when absorbed; therefore, electrons are pushed into the conduction band with excess kinetic energy, which is lost as heat. Accordingly, the development of new polymer nanocomposite materials with low band gap energy is essential for energy storage devices^36^.

DFT is one of the most useful and adaptable theories currently in use in this field of study. DFT has made it possible to understand chemical selectivity by looking at properties that are isolated from a compound, therefore making it easier to explain the reactivity of both simple and complex systems without needing to go into sophisticated details about the reaction pathway^12,37–39^. Additionally, DFT is the most accurate method, and the error in this computation is systematic, but it can be mitigated by using an experimental scale factor to bring the calculated findings closer to the experimental results. The future of these types of calculations is bright, as they include a wide range of scientific fields, including biology, physics, and many others. In this context, DFT has been used to derive a number of concepts and descriptors of chemical reactivity, including S, η, and ω. Moreover, μ in this theory can be utilized to characterize chemical reactivity locally^40,41^.

Scotland et al. reduced the bandgap energy of PANi to 1.300 eV by varying the benzoid–quinoid structural units and increasing oligomer length using the B3LYP/SV(P) model^42^. Meanwhile, the effect of hydration and nitrate and sulphate salts on the electronic properties of PANi was investigated by Farrage et al.^43^. The results showed that TDM increased to 10.1115 Debye and the bandgap energy decreased to 0.911 eV. Additionally, our group investigated the effect of a graphene oxide and teflon composite on the electronic properties of PANi. The results revealed that TDM increased to 5.839 Debye and the bandgap energy decreased to 0.268 eV^44^.

In this study, DFT was utilized to study the electronic, global reactivity descriptors, MESP, and vibrational characteristics of PANi, PANi/alkali metal oxide, and PANi/heavy metal oxide model molecules to develop new low band gap material suitable for energy storage devices. The frontier energy levels of conjugated polymers are critical factors for designing efficient energy storage materials. Thus, this work focused on investigating the following important reactivity descriptors: total dipole moment (TDM), highest occupied molecular orbital’s energy, lowest unoccupied molecular orbital’s energy, HOMO–LUMO band gap energy, IE, EA, η, μ, ω, S, χ, and MESP maps. A comprehensive comparison of the observed infrared (IR) bands is provided, and the assignments are based on IR intensities and atomic displacements of vibrational modes. Additionally, the IR intensities were calculated using different levels of theory for verification of the proposed model.

## Calculations details

Model molecules representing PANi, PANi/alkali metal oxide, and PANi/heavy metal oxide nanocomposites were subjected to optimization using Gaussian 09^45^ softcode which is implemented at the Molecular Modeling and Spectroscopy Laboratory, Centre of Excellence for Advanced Science, National Research Centre, Egypt. All molecules were modeled and optimized using the Stuttgart–Dresden (SDD) basis set at the B3LYP (Becke’s 3-parameter exchange functional with Lee–Yang–Parr correlation energy) level^46–48^. This B3LYP hybrid functional incorporates the exchange–correlation functional, which is based on the conventional version of the Vosko–Wilk–Nusair correlation potential^42^. The slater exchange was originally included in functional B, as were modifications to the density gradient. Lee, Yang, and Parr created a correlation functional LYP that includes both local and nonlocal terms^44,45^.

B3LYP is the most widely used DFT approach because it is capable of accurately predicting molecular structures and other properties. A hybrid functional called B3LYP was created in the late 1980s. It turns out that the recovery of electron correlation is the fundamental goal shared by DFT and Hartree–Fock based techniques. Though DFT has an exact form for dynamic electron correlation, it must approximate exchange correlation because it is not quantum mechanical. Hartree–Fock methods, on the other hand, precisely treat exchange correlation but struggle to recover dynamic electron correlation.

B3 is Becke’s three paramater exchange correlation functional, which uses three parameters to mix in the exact Hartree–Fock exchange correlation, and LYP is the Lee Yang and Parr correlation functional that recovers dynamic electron correlation. B3LYP is popular for many reasons. It was one of the first DFT methods that were a significant improvement over Hartree–Fock. B3LYP is generally faster than most Hartree–Fock approaches and usually yields comparable results. It is also fairly robust for a DFT method. On a more fundamental level, it is not as heavily parameterized as other hybrid functionals, having only three where some have up to 26^46^.

The PANi molecule interacted with one and two units of alkali and heavy metal oxides, respectively. TDM, HOMO–LUMO band gap energy, global reactivity descriptors, and MESP maps were calculated with the DFT: B3LYP/SDD model. HOMO and LUMO are keywords in quantum chemistry that reflect chemical bioactivity, according to frontier molecular orbital theory. The HOMO donates electrons, whereas the LUMO symbolizes the ability to gain an electron. As a result, studies on frontier molecular orbital (FMO) provide some important information on the active mechanism for physicists and chemists such as IE, EA, η, μ, ω, S, and χ known as global reactivity descriptors, which are calculated using the following equations^49^:

1 $${\text{IE}} = - {\text{E}}_{{{\text{HOMO}}}}$$

2 $${\text{IE}} = - {\text{E}}_{{{\text{LUMO}}}}$$

3 $$\Delta {\text{E}} = {\text{E}}_{{{\text{HOMO}}}} - {\text{E}}_{{{\text{LUMO}}}}$$

4 $$\eta = {{{\text{Energy}}\,{\text{gap}}} \mathord{\left/ {\vphantom {{{\text{Energy}}\,{\text{gap}}} 2}} \right. \kern-0pt} 2}$$

5 $$\mu = {{\left( {{\text{E}}_{{{\text{HOMO}}}} + {\text{E}}_{{{\text{LUMO}}}} } \right)} \mathord{\left/ {\vphantom {{\left( {{\text{E}}_{{{\text{HOMO}}}} + {\text{E}}_{{{\text{LUMO}}}} } \right)} 2}} \right. \kern-0pt} 2}$$

6 $$\omega = {{\mu^{2} } \mathord{\left/ {\vphantom {{\mu^{2} } {2\eta }}} \right. \kern-0pt} {2\eta }}$$

7 $$S = {1 \mathord{\left/ {\vphantom {1 \eta }} \right. \kern-0pt} \eta }$$

8 $$\upchi = - \mu$$

IE is simply defined as the energy needed to extract an electron from an isolated molecule^49^. Additionally, an attempt to verify the modeling results is achieved by comparing B3LYP/SSD with HF/6-31G(d), HF/6-31G(d,p), and B3LYP/6-31G(d,p) models. The IR frequencies for PANi were calculated with these models to be compared with the experimental FTIR spectrum of PANi.

## Results and discussions

### Building model molecules

**Fig. 1:**
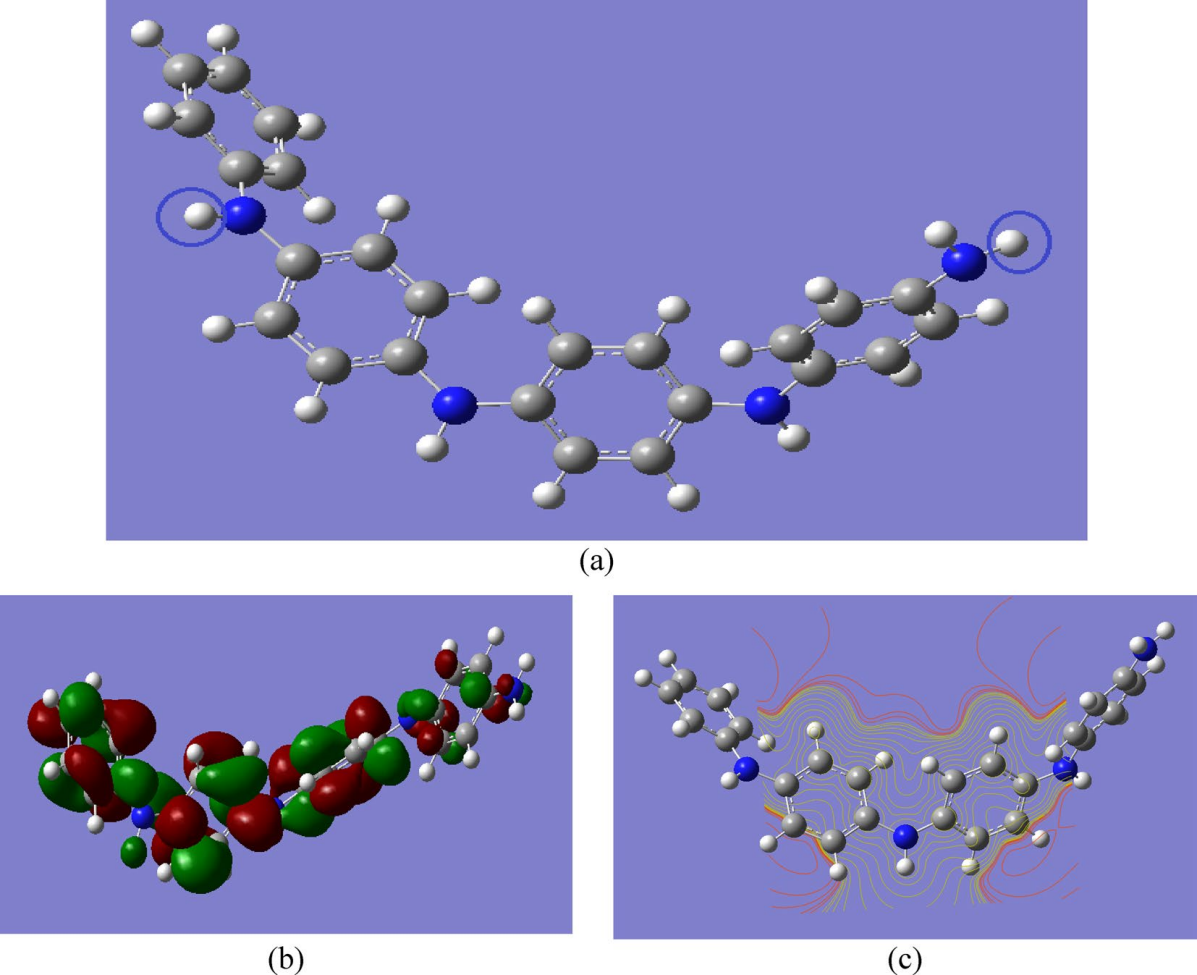
B3LYP/SSD (**a**) optimized structure of PANi consisting of four aniline units, where two hydrogen bonds of two amides in units 1 and 4; (**b**) HOMO–LUMO orbitals; (**c**) calculated MESP maps.

PANi is an intrinsically conducting polymer with many applications in wide areas of research. Therefore, a model molecule of PANi consisting of four aniline units is built. Functionalization of PANi with alkali metal oxides and heavy metal oxides was then carried out through the formation of two hydrogen bonds with two amide groups in the PANi structure (units 1 and 4), as presented in Fig. 1a. Alkali metal oxides such as lithium oxide (Li_2_O), sodium oxide (Na_2_O), potassium oxide (K_2_O), magnesium oxide (MgO), and calcium oxide (CaO) were chosen for functionalization of PANi. Additionally, heavy metal oxides such as CrO, manganese oxide (MnO), iron oxide (FeO and Fe_2_O_3_), cobalt oxide (CoO), nickel oxide (NiO), copper oxide (CuO), and zinc oxide (ZnO) were also studied.

### Frontier molecular orbitals analysis

HOMO and LUMO are two of the most significant parameters for quantum chemistry. These orbitals are also known as frontier molecular orbitals (FMO) because they are located at the outermost borders of the electrons in molecules. The magnitude of the HOMO–LUMO band gap energy directly impacts the short circuit current in organic solar cells; the HOMO energy of the electron donor relative to the LUMO energy of the electron acceptor is proportional to the open-circuit voltage. The efficacy of the different sensors is often related to the FMO. Because the interaction of the FMO of PANi and metal oxides is expected to alter the charge transport and optical properties of the systems, allowing the detection of gases, estimating the distribution of the FMO of the nanocomposites is extremely relevant in order to identify new potential systems^50^.

The HOMO–LUMO molecular orbital distribution plot of PANi is illustrated in Fig. 1b, while Fig. 1c presents the MESP map of PANi. Since it is a measure of electron conductivity, the energy gap between HOMO and LUMO is a crucial metric in understanding the parameters of molecular electrical transport. The ability to receive an electron is represented by the LUMO as an electron acceptor, while the ability to give an electron is represented by the HOMO^50–52^. Also, the TDM, which measures the polarization of the studied structures, was calculated. The HOMO–LUMO band gap energy and TDM of PANi are determined to be 4.044 eV and 2.926 Debye, respectively.

### Functionalizantion of PANi with alkali metal oxides

**Fig. 2:**
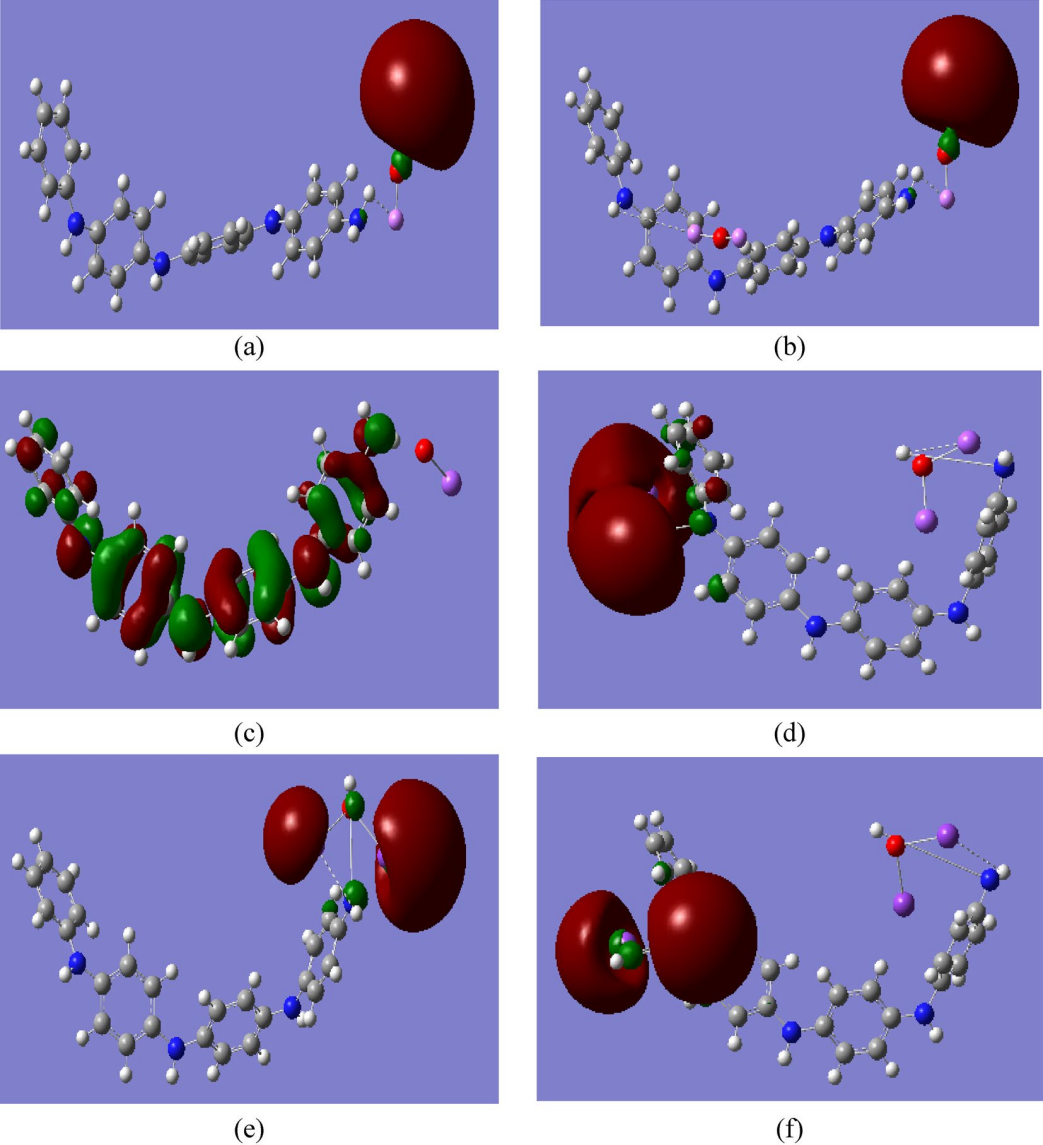

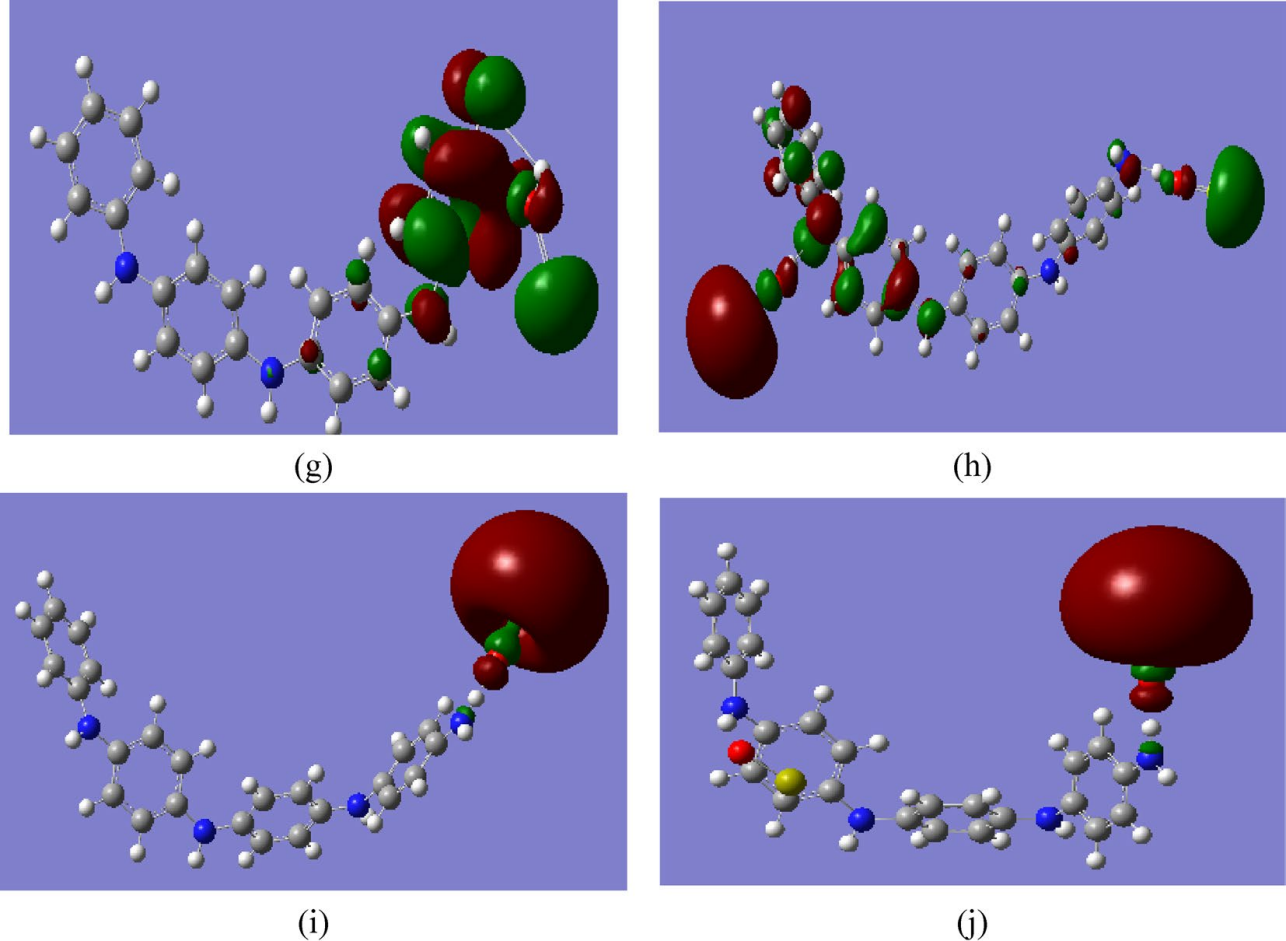
B3LYP/SSD calculated HOMO/LUMO orbitals of PANi functionalized with (**a**) Li_2_O, (**b**) 2Li_2_O, (**c**) Na_2_O, (**d**) 2Na_2_O, (**e**) K_2_O, (**f**) 2K_2_O, (**g**) MgO, (**h**) 2MgO, (**i**) CaO, and (**j**) 2CaO.

To understand the effect of the alkali metal oxides on the electronic properties of PANi, the changes induced on the electronic gaps (ΔE = E_LUMO_ − E_HOMO_), relative to the unmodified PANi were illustrated in Fig. 2 for PANi functionalized with one and two molecules of Li_2_O, Na_2_O, K_2_O, MgO, and CaO. The figures show that the electronic charges are redistributed within the PANi structure due to functionalization with Li_2_O, Na_2_O, K_2_O, MgO, and CaO.

In general, significant differences are observed for the majority of the systems. In particular, PANi-NaO_2_, PANi-MgO, and PANi-CaO present higher chemical stabilities, especially PANi-CaO, with ΔE lower than 1 eV. Table 1 presents the changes in the ΔE of PANi as a result of functionalization. The table shows that the ΔE of PANi decreased from 4.044 eV to 3.203, 1.551, 2.970, 1.533, and 0.846 eV due to functionalization with one molecule of LiO_2_, NaO_2_, KO_2_, MgO, and CaO, respectively.

**Table 1:** B3LYP/SDD calculated total dipole moment (TDM) as Debye; HOMO–LUMO band gap energy (∆E) as eV for PANi functionalized with alkali metal oxides.

Structure	TDM (Debye)	∆E (eV)
PANi	2.926	4.044
PANi–Li_2_O	4.200	3.203
PANi–2Li_2_O	3.782	2.749
PANi–Na_2_O	13.483	1.551
PANi–2Na_2_O	9.560	3.074
PANi–K_2_O	6.850	2.970
PANi–2K_2_O	5.208	2.789
PANi–MgO	4.219	1.533
PANi–2MgO	5.089	0.465
PANi–CaO	14.895	0.846
PANi–2CaO	16.439	1.098

Additionally, increasing the number of alkali metal oxide units had a significant impact on the ΔE of PANi. Functionalization of PANi with 2LiO_2_, 2NaO_2_, 2KO_2_, 2MgO, and 2CaO functional groups has major impacts on the PANi’s electronic properties, especially for PANi-2MgO, where the ΔE exhibited the same quantitative trend and decreased to 0.465 eV. When compared to pristine PANi, all of the systems have smaller electronic gaps, with prominent variations in the case of 1MgO, 2MgO, and 1CaO groups.

Table 1 presents the TDM values of PANi and functionalized PANi. TDM increased as a result of interaction with alkali metal oxides, indicating that functionalized PANi is more polarizable than pristine PANi, where TDM increased from 2.9259 Debye to 4.200, 13.483, 6.850, 4.219, and 14.895 Debye for PANi functionalized with LiO_2_, NaO_2_, KO_2_, MgO, and CaO, respectively. Moreover, increasing the number of functional groups to two units did not have a strong impact on TDM, as it increased to 3.782, 5.208, and 5.089 Debye in the case of 2LiO_2_, 2KO_2_, and 2MgO, respectively. However, functionalization of PANi with 2NaO_2_ and 2CaO functional groups had a stronger impact on its properties as TDM increased to 9.560 and 16.439 Debye, respectively. Finally, the interaction between PANi and alkali metal oxides, particularly MgO and CaO functional groups, leads to the conclusion that PANi may be more conductive.

Descriptors of global chemical reactivity are used to comprehend the link between compound structure, stability, and global chemical reactivity. The estimated global reactivity descriptors of PANi functionalized with alkali metal oxides for the B3LYP/SSD were calculated and listed in Table 2

**Table 2:** The B3LYP/SSD calculated global reactivity descriptors of PANi functionalized with alkali metal oxides.

Structure	HOMO (eV)	LUMO (eV)	IE (eV)	EA (eV)	η (eV)	µ (eV)	ω (eV)	S (eV)^−1^	χ (eV)
PANi	− 4.327	− 0.283	4.327	0.283	2.022	− 2.305	1.314	0.495	2.305
PANi–Li_2_O	− 4.269	− 1.066	4.269	1.066	1.601	− 2.668	2.222	0.625	2.668
PANi–2Li_2_O	− 3.804	− 1.055	3.804	1.055	1.375	− 2.430	2.147	0.727	2.430
PANi–Na_2_O	− 3.942	− 2.389	3.942	2.389	0.776	− 3.166	6.455	1.288	3.166
PANi–2Na_2_O	− 4.029	− 0.954	4.029	0.954	1.537	− 2.492	2.020	0.651	2.492
PANi–K_2_O	− 3.961	− 0.992	3.961	0.992	1.485	− 2.477	2.065	0.673	2.477
PANi–2K_2_O	− 3.673	− 0.884	3.673	0.884	1.394	− 2.279	1.862	0.717	2.279
PANi–MgO	− 3.970	− 2.437	3.970	2.437	0.766	− 3.203	6.694	1.304	3.204
PANi–2MgO	− 4.109	− 3.645	4.109	3.645	0.232	− 3.877	32.363	4.306	3.877
PANi–CaO	− 3.803	− 2.957	3.803	2.957	0.423	− 3.380	13.502	2.364	3.380
PANi–2CaO	− 4.159	− 3.061	4.159	3.061	0.549	− 3.610	11.867	1.822	3.610

The obtained values of IE indicated that removing an electron from HOMO to LUMO follows the order: PANi > PANi/Li_2_O > PANi/2CaO > PANi/2MgO > PANi/2Na_2_O > PANi/MgO > PANi/K_2_O > PANi/Na_2_O > PANi/2Li_2_O > PANi/CaO > PANi/2K_2_Oas shown in Table 2.

The energy change of an atom that is neutral (in the gaseous phase) when an electron has been added to the atom to form a negative ion is known as EA. In other words, it is defined as the probability of a neutral atom to gain an electron. First EAs, or negative affinities, result from the release of energy when an electron is added to a neutral atom. Second affinities are positive because the energy needed to add an electron to a negative ion (i.e., second EA) exceeds the energy released during the electron attachment process^53^. EA follows the order: PANi < PANi/2K_2_O < PANi/2Na_2_O < PANi/K_2_O < PANi/2Li_2_O < PANi/Li_2_O < PANi/Na_2_O < PANi/MgO < PANi/CaO < PANi/2CaO < PANi/2MgO as shown in Table 2.

The ability of charge transfer within the molecule is reflected in the results of small η values for the structures under study^53^. Consequently, η of PANi functionalized with alkali metal oxides follows the sequence: PANi > PANi/Li_2_O > PANi/2Na_2_O > PANi/K_2_O > PANi/2K_2_O > PANi/2Li_2_O > PANi/Na_2_O > PANi/MgO > PANi/2CaO > PANi/CaO > PANi/2MgO for charge transfer inside the molecule. Tables1 and 2 illustrate the linear relationship between η and HOMO–LUMO band gap energy. When it comes to η values, the harder the molecule, the higher the η values, the lower the charge transfer and vice versa^54^.

“μ” represents the tendency of an electron to escape from a stable system. The results show that μ of PANi decreased due to functionalization with alkali metal oxides and follow the sequence: PANi/2K_2_O > PANi > PANi/2Li_2_O > PANi/K_2_O > PANi/2Na_2_O > PANi/Li_2_O > PANi/Na_2_O > PANi/MgO > PANi/1CaO > PANi/2CaO > PANi/2MgO, as shown in Table 2.

The tendency of a system to absorb electrons is known as the electrophilicity index (ω)^53^. The ω values have increased and the μ values have reduced due to functionalization. As the molecules with low μ and high ω values have a good electrophile character, it can be concluded that model molecules representing PANi- 2MgO is the most electrophilic structure.

Table 2 shows that, due to functionalization of PANi with alkali metal oxides, the η values have reduced and the S values have increased. Intramolecular charge transfer is more likely to occur in the model molecule representing PANi/2MgO, as it has a lower η and a higher S value in the gaseous phase than other model molecules. The lower S value of PANi (0.495 eV^−1^) confirms that it is a less polarizable molecule.

The negative value of μ is known as χ^52,54–56^. The variation of χ values is supported by electrostatic potential, for any two molecules, where electrons will be partially transferred from one of low χ to that of high χ. The results show that the order of decreasing χ is: PANi/2MgO > PANi/CaO > PANi/2CaO > PANi/MgO > PANi/Na_2_O > PANi/2Li_2_O > PANi/2K_2_O > PANi/K_2_O > PANi/2Na_2_O > PANi/Li_2_O > PANi as presented in Table 2.

### Functionalizantion of PANi with heavy metal oxides

**Fig. 3:**
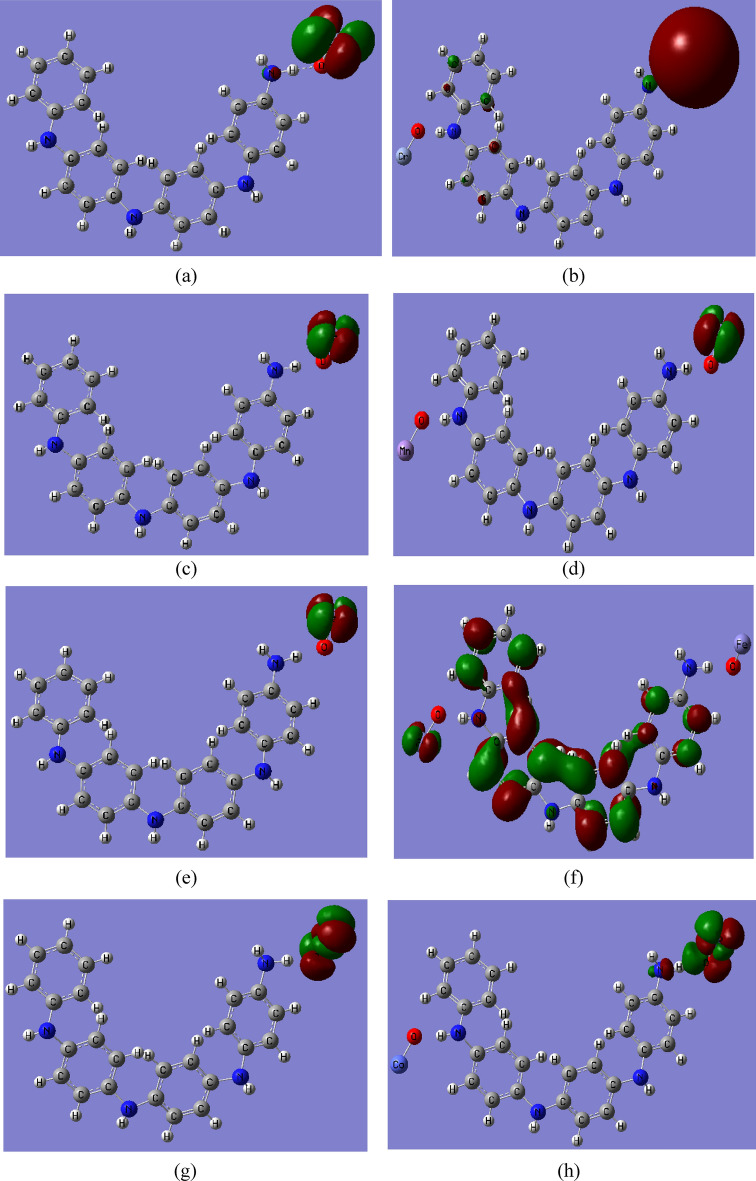

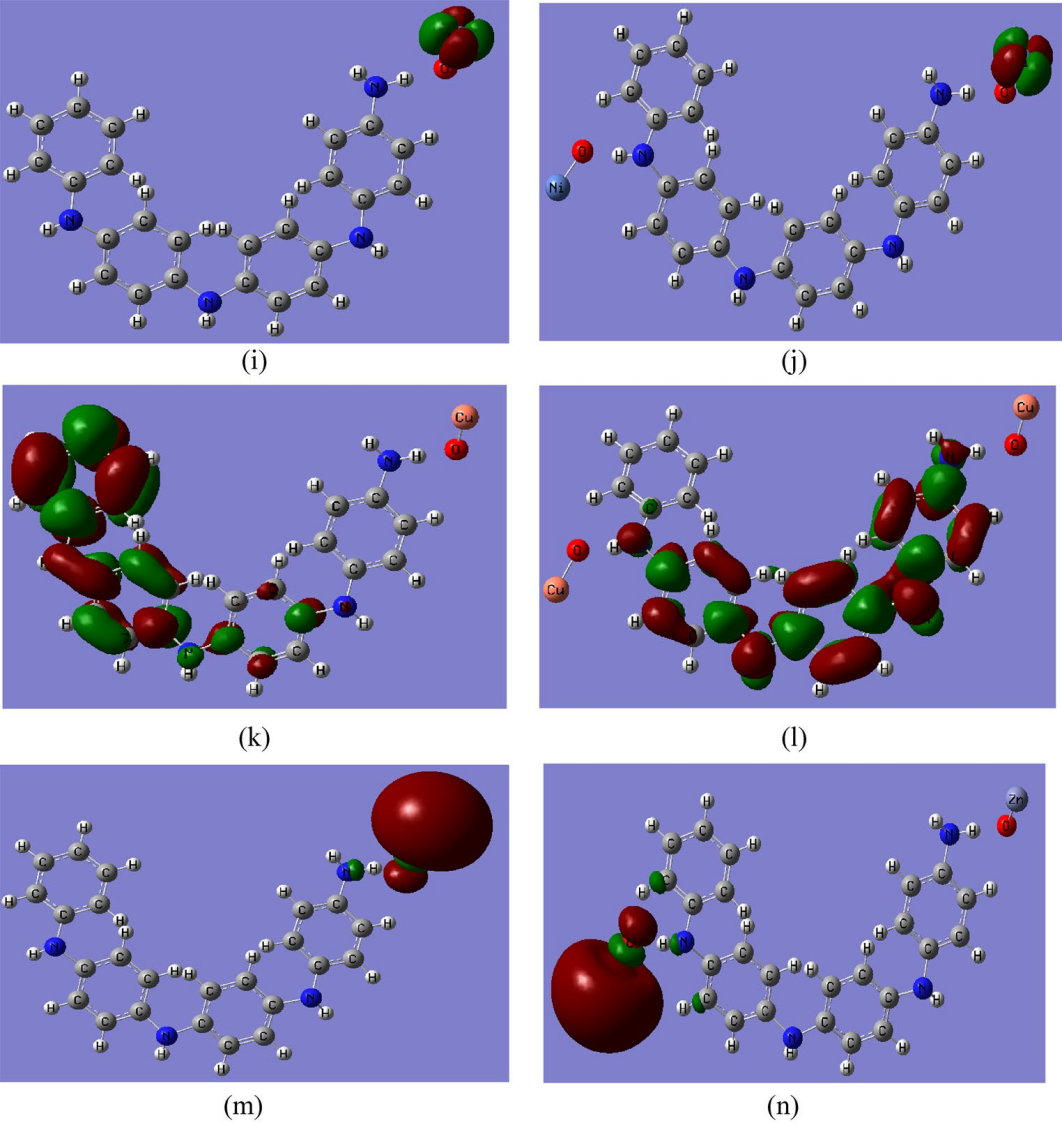
B3LYP/SSD calculated HOMO/LUMO orbitals of PANi functionalized with (**a**) 1 CrO, (**b**) 2 CrO, (**c**) MnO, (**d**) 2MnO, (**e**) FeO, (**f**) 2FeO, (**g**) CoO, (**h**) 2CoO, (**i**) NiO, (**j**) 2NiO, (**k**) CuO, (**l**) 2CuO, (**m**) ZnO, and (**n**) 2ZnO.

On the other hand, for PANi functionalized with the heavy metal oxides CrO, MnO, FeO, CoO, NiO, CuO, and ZnO, TDM and ΔE were also calculated. Table 3 presents the changes in the ΔE and TDM of PANi as a result of functionalization with heavy metal oxides. Figure 3 presented the mapped HOMO/LUMO orbitals of PANi functionalized with the studied heavy metals.

**Table 3:** B3LYP/SDD calculated total dipole moment (TDM) as Debye and HOMO–LUMO band gap energy (∆E) as eV for PANi functionalized with heavy metal oxides.

Structure	TDM (Debye)	∆E (eV)
PANi	2.926	4.044
PANi–CrO	3.090	1.135
PANi–2CrO	1.220	0.929
PANi–MnO	1.295	1.025
PANi–2MnO	2.681	0.196
PANi–FeO	3.872	1.248
PANi–2FeO	3.015	0.518
PANi–CoO	1.931	0.655
PANi–2CoO	3.386	0.207
PANi–NiO	1.274	1.053
PANi–2NiO	2.411	0.955
PANi–CuO	1.188	1.370
PANi–2CuO	5.129	0.266
PANi–ZnO	1.708	0.727
PANi–2ZnO	1.816	0.674

The ΔE of PANi decreased from 4.044 eV to 1.135, 1.025, 1.248, 0.655, 1.053, 1.370, and 0.727 eV due to functionalization with one molecule of CrO, MnO, FeO, CoO, NiO, CuO, and ZnO, respectively. The TDM of PANi changed to 3.090, 1.295, 3.872, 1.931, 1.274, 1.188, and 1.708 Debye due to functionalization with one molecule of CrO, MnO, FeO, CoO, NiO, CuO, and ZnO, respectively. These changes in the TDM value of PANi as a result of functionalization with heavy metal oxides confirm the formation of hydrogen bonding between the hydrogen atom of PANi and the oxygen atom of the studied heavy metals. The TDM of PANi changed to 1.220, 2.681, 3.015, 3.386, 2.411, 5.129, and 1.816 Debye, and ΔE decreased to 0.929, 0.196, 0.518, 0.207, 0.955, 0.266, and 0.674 eV for PANi functionalized with 2CrO, 2MnO, 2FeO, 2CoO, 2NiO, 2CuO, and 2ZnO, respectively. Therefore, it can be concluded that functionalization of PANi with two units of heavy metal oxides has a strong impact on the electronic properties of PANi, thus dedicating PANi to different applications.

Table 4 presents the changes in the global reactivity descriptors calculated at the B3LYP/SSD level of theory as a result of the change in both HOMO and LUMO energies of PANi due to functionalization with heavy metal oxides, confirming the change in the physical characteristics of PANi. Table 4 presents that IE increased from 4.327 eV to 4.809, 4.860, 4.755, 4.793, 5.102, 5.179, and 4.824 eV due to functionalization with CrO, MnO, FeO, CoO, NiO, CuO, and ZnO, respectively, and to 4.796, 4.774, 4.729, 4.874, 5.221, 5.091, and 4.796 eV due to functionalization with 2CrO, 2MnO, 2FeO, 2CoO, 2NiO, 2CuO, and 2ZnO, respectively.

**Table 4:** The B3LYP/SSD calculated global reactivity descriptors of PANi functionalized with heavy metal oxides.

Structure	HOMO (eV)	LUMO (eV)	IE (eV)	EA (eV)	η (eV)	μ (eV)	ω (eV)	S (eV)^−1^	χ (eV)
PANi	− 4.327	− 0.283	4.327	0.283	2.022	− 2.305	1.314	0.495	2.305
PANi–CrO	− 4.809	− 3.674	4.809	3.674	0.568	− 4.242	15.850	1.762	4.242
PANi–2CrO	− 4.796	− 4.122	4.796	4.122	0.337	− 4.459	29.499	2.967	4.459
PANi–MnO	− 4.860	− 3.835	4.860	3.835	0.513	− 4.348	18.439	1.951	4.348
PANi–2MnO	− 4.774	− 4.578	4.774	4.578	0.098	− 4.676	111.556	10.208	4.676
PANi–1FeO	− 4.755	− 3.507	4.755	3.507	0.624	− 4.131	13.674	1.602	4.131
PANi–2FeO	− 4.729	− 4.210	4.729	4.210	0.259	− 4.469	38.490	3.858	4.469
PANi–CoO	− 4.793	− 4.138	4.793	4.138	0.328	− 4.466	30.443	3.052	4.466
PANi–2CoO	− 4.874	− 4.667	4.874	4.667	0.103	− 4.771	109.940	9.684	4.772
PANi–1NiO	− 5.102	− 4.049	5.102	4.049	0.526	− 4.575	19.881	1.900	4.575
PANi–2NiO	− 5.221	− 4.266	5.221	4.266	0.478	− 4.744	23.561	2.094	4.744
PANi–1CuO	− 5.179	− 2.640	5.179	2.640	1.270	− 3.909	6.0197	0.788	3.909
PANi–2CuO	− 5.091	− 4.825	5.091	4.825	0.133	− 4.958	92.412	7.531	4.958
PANi–1ZnO	− 4.823	− 4.096	4.824	4.096	0.364	− 4.460	27.355	2.751	4.460
PANi–2ZnO	− 4.796	− 4.122	4.796	4.122	0.337	− 4.459	29.499	2.967	4.459

Meanwhile, Table 4 showed that increasing the number of heavy metal oxide molecules causes the EA to increase. EA increased from 3.674, 3.835, 3.507, 4.138, 4.049, 2.640, and 4.096 eV for CrO, MnO, FeO, CoO, NiO, CuO, and ZnO, respectively, to 4.122, 4.578, 4.210, 4.667, 4.266, 4.825, and 4.122 eV for 2CrO, 2MnO, 2FeO, 2CoO, 2NiO, 2CuO, and 2ZnO, respectively. The highest value of EA belongs to the structure representing PANi/2CuO.The opposite was observed for η, which decreased with increasing the number of heavy metal oxide molecules to two, reaching 0.337, 0.098, 0.259, 0.103, 0.478, 0.133, and 0.337 eV for PANi functionalized with 2CrO, 2MnO, 2FeO, 2CoO, 2NiO, 2CuO, and 2ZnO, respectively, instead of 0.568, 0.513, 0.624, 0.328, 0.526, 1.270, and 0.364 eV for PANi functionalized with CrO, MnO, FeO, CoO, NiO, CuO, and ZnO, respectively. This means that the resistance to redistribute electrons within the PANi structure decreased, thus reflecting the higher reactivity of the structure. Meanwhile, μ of PANi decreased from − 2.305 eV to − 4.242, − 4.348, − 4.131, − 4.466, − 4.575, − 3.909, and − 4.460 eV for PANi functionalized with CrO, MnO, FeO, CoO, NiO, CuO, and ZnO, respectively, and decreased to − 4.459, − 4.676, − 4.469, − 4.771, − 4.744, − 4.958, and − 4.459 Debye for PANi functionalized with 2CrO, 2MnO, 2FeO, 2CoO, 2NiO, 2CuO, and 2ZnO, respectively. These changes mean that the tendency of electrons to escape decreased with increasing the number of molecules of the heavy metal oxides. The lowest value of μ (−4.958 eV) belongs to the structure representing PANi/2CuO. Meanwhile, the value of ω increased from 15.850, 18.439, 13.674, 30.443, 19.881, 6.0197, and 27.355 eV for CrO, MnO, FeO, CoO, NiO, CuO, and ZnO, respectively, to 29.499, 111.556, 38.490, 109.94, 23.561, 92.412, and 29.499 eV for 2CrO, 2MnO, 2FeO, 2CoO, 2NiO, 2CuO, and 2ZnO, respectively, indicating increased electrophilicity, thus increased reactivity, with increasing the number of molecules of CrO, MnO, FeO, CoO, NiO, CuO, and ZnO from one to two. Finally, Table 4 shows that both S and χ increased with increasing the number of molecules of the heavy metal oxides.

### Molecular electrostatic potential analysis

**Fig. 4:**
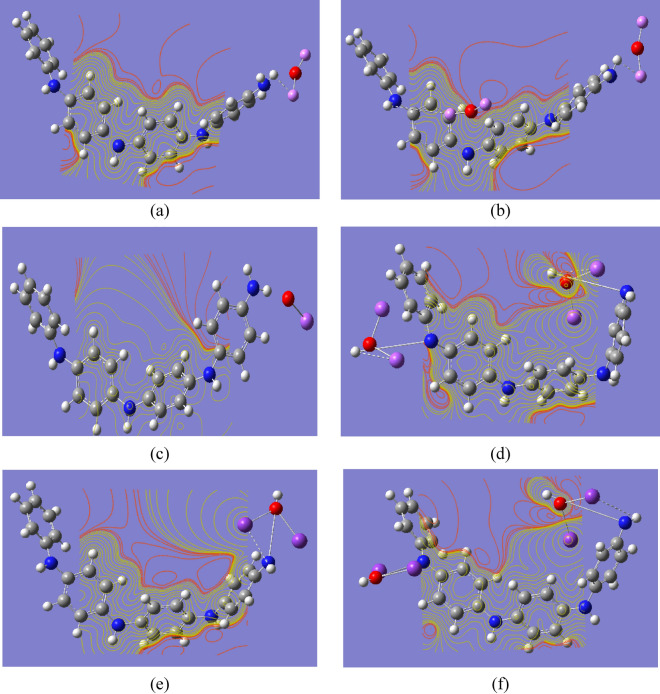

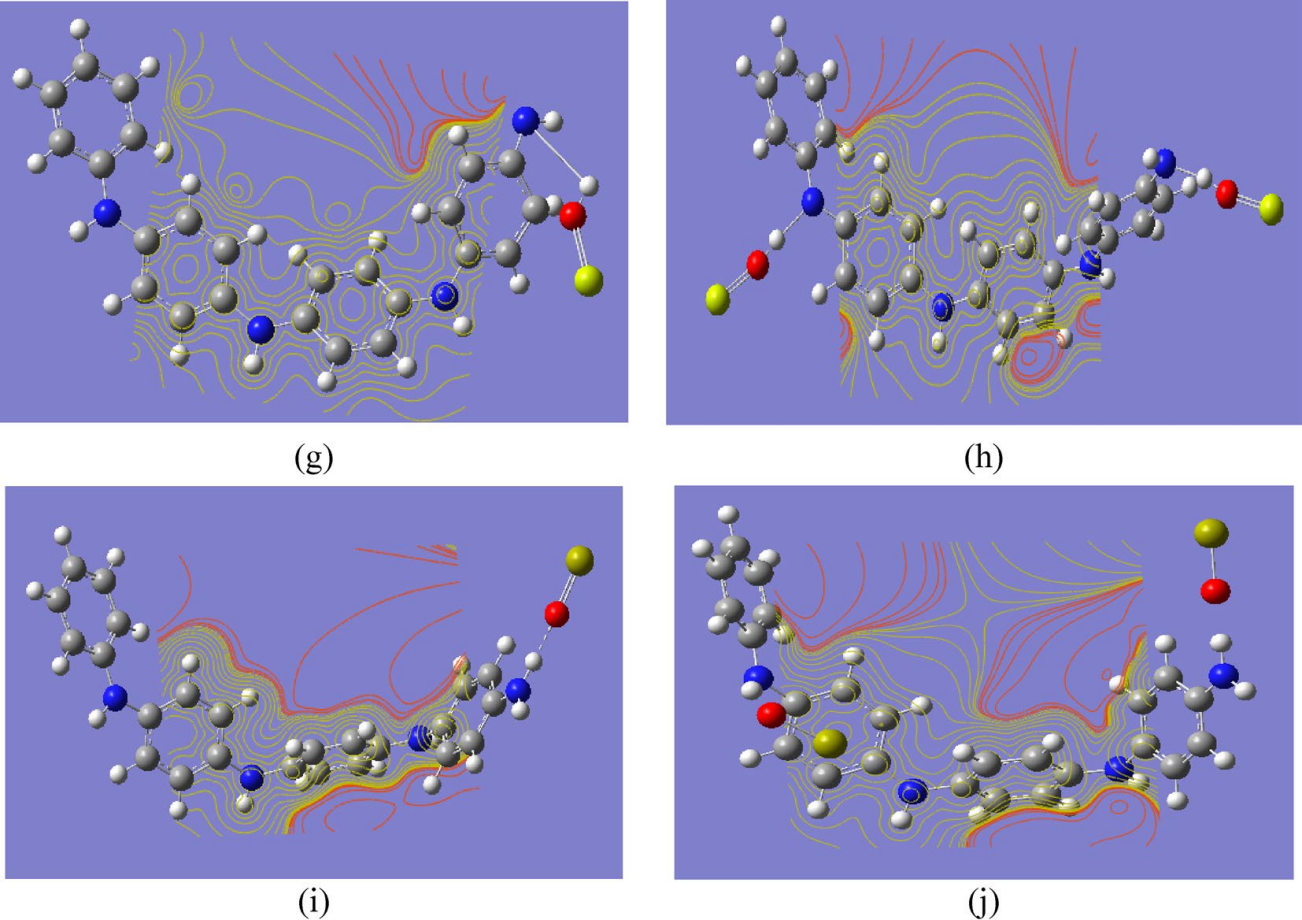
B3LYP/SSD calculated MESP contour maps of PANi functionalized with (**a**) Li_2_O, (**b**) 2Li_2_O, (**c**) Na_2_O, (**d**) 2Na_2_O, (**e**) K_2_O, (**f**) 2K_2_O, (**g**) MgO, (**h**) 2MgO, (**i**) CaO, and (**j**) 2CaO.

**Fig. 5:**
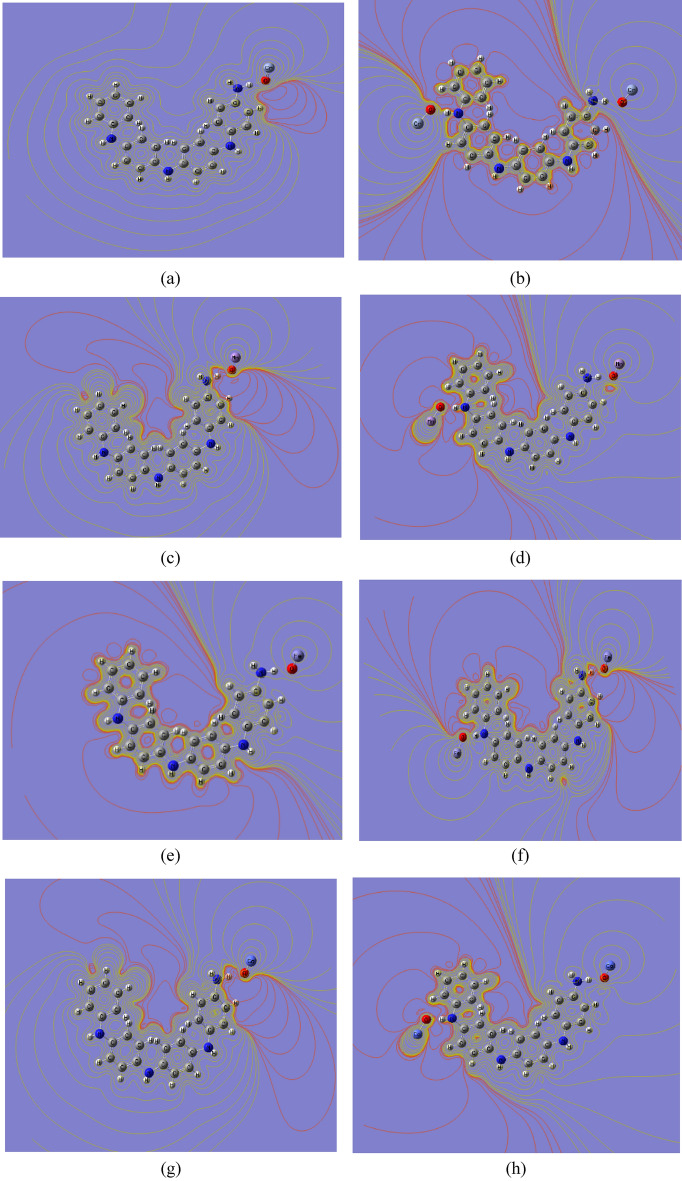

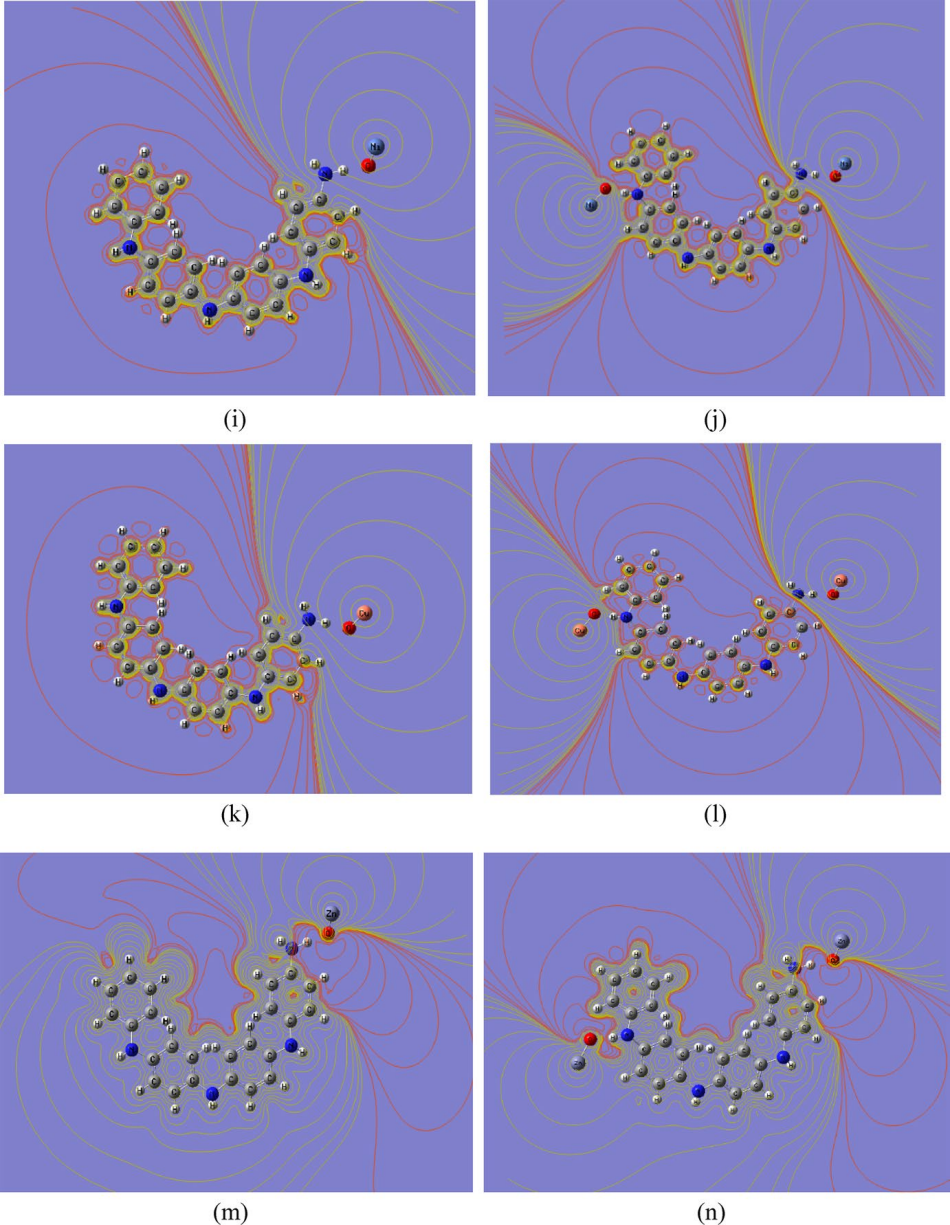
B3LYP/SSD calculated MESP contour of PANi functionalized with (**a**) 1 CrO, (**b**) 2 CrO, (**c**) MnO, (**d**) 2MnO, (**e**) FeO, (**f**) 2FeO, (**g**) CoO, (**h**) 2CoO, (**i**) NiO, (**j**) 2NiO, (**k**) CuO, (**l**) 2CuO, (**m**) ZnO, and (**n**) 2ZnO.

MESP is used to characterize inter- and intramolecular electrostatic interactions. The charge distribution on the surface can be utilized to determine both the interactions of active molecules and the sort of chemical bond. In structural biology, MESP is required to determine drug-receptor interactions, ligand-substrate interactions, and enzyme–substrate interactions^57,58^. Additionally, MESP is used to predict reactive molecular locations. Figure 1c depicts the MESP contour map of emeraldine base PANi. Different colors indicate different electrostatic potential levels on the contour map. Electrophilic interaction is represented by the red color region (negative electrostatic potential), while nucleophilic attack is represented by the blue color region (positive electrostatic potential)^57^. The electronegativity increases in the following order: red > orange > yellow > green > blue^58^. As shown in the figure, the negative area is mostly concentrated around the nitrogen atom, while the sites for nucleophilic attack occur around the hydrogen atoms. Figures 4 and 5 show the B3LYP/SSD calculated MESP contour maps for PANi functionalized with alkali and heavy metal oxides, respectively. It is clear from the figure that the functionalization of PANi with alkali metal oxides causes the MESP maps to change over the whole PANi’s structure. As presented in Figs. 4 and 5, the intensity of the red color increased as a result of functionalization of PANi with alkali and heavy metal oxides. Figures showed that the electrons are redistributed along the PANi’s chain. Additionally, increasing the units of the metal oxides causes the electronegativity to increase further following the sequence: PANi-2MgO > PANi-1CaO > PANi-2CaO > PANi-1 NaO_2_ > PANi-2LiO_2_ > PANi-2KO_2_. However, for PANi functionalized with heavy metal oxides, Fig. 5 showed that functionalization of PANi with heavy metal oxides has a stronger impact on PANi’s electronic characteristics than that with alkali metal oxides. As presented in Fig. 5, almost all the studied structures of functionalized PANi possess superior reactivity, as the intensity of the red color increased strongly, and the negative charges became distributed throughout the whole structure. Moreover, the figure showed that the electronegativity of PANi-1MnO, PANi-2MnO, PANi-1CuO, and PANi-2CuO is higher than that of the other molecules. The obtained results of MESP are in good agreement with those of the TDM, HOMO–LUMO band gap energy, and global reactivity descriptors. It can, therefore, be concluded that PANi functionalized with heavy metal oxides are suitable candidates to be used in energy storage devices.

### Accuracy of the model

To verify the studied model, the vibrational spectrum of PANi is compared with experimental FTIR results. The FTIR frequencies of PANi are presented in Table 5, and the assignment is aided by the previous work^59,60^. As shown in Table 5, rings 1, 2, and 3 have a band at 1573 cm^−1^. Then, the C = N band of ring 2 has two respective bands at 1659 cm^−1^ and 1599 cm^−1^. The C–N band of ring 4 is located at 1531 cm^−1^. The band at 1493 cm^−1^ is corresponding to the C–N of ring 3, then ring 4 has a band at 1341 cm^−1^. The C–N band at 1285 cm^−1^ is assigned for rings 3 and 4. The last band, which appeared at 1166 cm^−1^, is assigned to rings 1, 2, 3, and 4. Comparing the experimental FTIR results with the calculated IR by the four models in Table 5, it is clear that the B3LYP/SSD model is comparable with other levels of theory, and also gives good agreement with the experimental FTIR results. Any differences between the observed and predicted wave numbers are due to the fact that the calculations were performed on a single (or isolated) molecule in its gaseous state. This could be an indication for the suitability of applying the B3LYP/SSD model to study PANi for the parameters obtained with both first and second derivatives.

**Table 5:** The B3LYP/SSD calculated IR spectrum of PANi as compared with HF/6-31G(d), HF/6-31G(d,p), B3LYP/6-31G(d,p) and experimental FTIR spectrum.

FTIR	Band assignment	HF		B3LYP	
		6-31G(d)	6-31G(d,p)	SDD	6-31G(d,p)
1659	Ring 2; C = N	1647	1690	1638	1663
1599	Ring 2; C = N	1599	1597	1565	1573
1573	Ring 1, 3, 4	1576	1547	1552	1559
1531	Ring 4; C–N	1500	1540	1545	1535
1493	Ring 3; C–N	1490	1438	1526	1499
1341	Ring 4	1373	1391	1347	1353
1285	Ring 3, 4; C–N	1259	1285	1293	1275
1166	Ring 1, 2, 3, 4	1186	1195	1162	1257

To evaluate and verify the energy calculations performed using SDD basis set, further verification of the energy values of PANI was also performed using HF/6-31G(d), HF/6-31G(d,p) and DFT/6-31G(d,p). The obtained results are listed in Table 6. As seen from the results in the table, the energy values obtained SDD and 6-31 g(d,p) basis sets of DFT method are comparable.

**Table 6:** The B3LYP/SSD calculated total energy, HOMO, LUMO and HOMO–LUMO band gap energy (∆E) as eV of PANi as compared with the values calculated using HF/6-31G(d,p) and B3LYP/6-31G(d,p).

	B3LYP/SDD	HF/6-31G(d,p)	B3LYP/6-31 g(d,p)
Total Energy (eV)	− 31,201.688	− 31,006.936	− 31,207.348
HOMO (eV)	− 4.316	− 7.484	− 4.222
LUMO (eV)	− 0.308	3.572	− 0.075
ΔE (eV)	4.044	11.056	4.147

The effect of basis set superposition error (BSSE) on the geometries optimized using SDD basis set has also been examined by calculating the BSSE of PANi–1NiO as an example at the same level of theory (with counterpoise = 2). The counterpoise-corrected energy of PANi–1NiO interaction was −37,878.573 eV, compared to −37,833.510 eV for the uncorrected one, with the BSSE energy of 0.077 eV. The value of the BSSE energy and the difference between the counterpoise-corrected and uncorrected total energies is negligibly small owing to the high basis set used, suggesting that the re-optimization of the structures based on the counterpoise-corrected potential energy surface is not crucial to compute the binding energy^61^.

## Conclusion

In this study, an attempt has been made to evaluate how different alkali metal oxides and heavy metal oxides affect the stability, electronic properties, and reactivity of PANi. Accordingly, emeraldine-base PANi was functionalized with the alkali metal oxides Li_2_O, Na_2_O, K_2_O, MgO, and CaO and the heavy metal oxides CrO, MnO, FeO, CoO, NiO, CuO, and ZnO. All calculations were carried out utilizing DFT at the B3LYP/SSD level of theory. The results showed that TDM of PANi increased and ΔE decreased due to functionalization. ΔE determines the significant degree of charge transfer interactions occurring in the compound and is also responsible for the molecule’s enhanced chemical activity. The results show that η and μ decreased due to functionalization, while EA, ω, S, and χ increased. Moreover, the results showed that functionalization of PANi with 2MgO and 2MnO strongly influenced the reactivity of PANi, as the value of ω increased from 1.314 eV to 32.363 and 111.556 eV, respectively. According to reactivity predictions, the MESP map clearly showed that the possible sites for electrophilic attack are primarily located over oxygen and nitrogen atoms, while the sites for nucleophilic attack are located around hydrogen atoms. Additionally, the results showed that the electrophilic ability of PANi increased while the nucleophilic ability decreased due to functionalization. In terms of calculated and measured IR frequencies of PANi, the B3LYP/SSD model showed comparable results in comparison with those obtained experimentally, as well as both HF and DFT methods. The obtained results reflect the strong reactivity of PANi–2MgO and PANi–2MnO that dedicates these molecules for energy storage devices.

## Data Availability

The data will be available upon request. Contact Medhat A. Ibrahim: ma.khalek@nrc.sci.eg.
